# Supplementary material for: HIVprotI: an integrated web based platform for prediction and design of HIV proteins inhibitors
Source: J Cheminform. 2018 Mar 9;10:12. doi: 10.1186/s13321-018-0266-y (PMC5845081; doi:10.1186/s13321-018-0266-y)
Supplement: Supplementary file 2 — Additional file 2. Source code of HIVProtI web server. [file 13321_2018_266_MOESM2_ESM.zip › HIVprotI_Source-code/hivprotI/weka-3-6-6/doc/weka/associations/RuleGeneration.html]

RuleGeneration


---


|  |  |  |
| --- | --- | --- |
| **Overview** \| **Package** \| **Class** \| **Tree** \| **Deprecated** \| **Index** \| **Help** \| **Weka's home** | |  |
| **PREV CLASS**   **NEXT CLASS** | **FRAMES**    **NO FRAMES**     **All Classes** |  |
| SUMMARY: NESTED \| FIELD \| CONSTR \| METHOD | DETAIL: FIELD \| CONSTR \| METHOD |  |


---


## weka.associations Class RuleGeneration

```
java.lang.Object
  weka.associations.RuleGeneration
```

**All Implemented Interfaces:**: java.io.Serializable, RevisionHandler

**Direct Known Subclasses:**: CaRuleGeneration

---

``` public class RuleGeneration extends java.lang.Object implements java.io.Serializable, RevisionHandler ```

Class implementing the rule generation procedure of the predictive apriori algorithm.
Reference: T. Scheffer (2001). *Finding Association Rules That Trade Support
Optimally against Confidence*. Proc of the 5th European Conf.
on Principles and Practice of Knowledge Discovery in Databases (PKDD'01),
pp. 424-435. Freiburg, Germany: Springer-Verlag.

The implementation follows the paper expect for adding a rule to the output of the
*n* best rules. A rule is added if:
the expected predictive accuracy of this rule is among the *n* best and it is
not subsumed by a rule with at least the same expected predictive accuracy
(out of an unpublished manuscript from T. Scheffer).

**Version:**
:   $Revision: 1.4 $

**Author:**
:   Stefan Mutter (mutter@cs.waikato.ac.nz)

**See Also:**: Serialized Form

---

**Constructor Summary**

`RuleGeneration(ItemSet itemSet)`
  
          Constructor


| **Method Summary** | |
| --- | --- |
| `static boolean` | `aSubsumesB(RuleItem a, RuleItem b)`             Methods that decides whether or not rule a subsumes rule b. |
| `static double` | `binomialDistribution(double accuracy, double ruleCount, double premiseCount)`             calculates the probability using a binomial distribution. |
| `boolean` | `change()`             Gets if the list fo the best rules has been changed |
| `int` | `count()`             Gets the actual maximum value of the generation time |
| `static double` | `expectation(double ruleCount, int premiseCount, double[] midPoints, java.util.Hashtable priors)`             calculates the expected predctive accuracy of a rule |
| `java.util.TreeSet` | `generateRules(int numRules, double[] midPoints, java.util.Hashtable priors, double expectation, Instances instances, java.util.TreeSet best, int genTime)`             Generates all rules for an item set. |
| `java.lang.String` | `getRevision()`             Returns the revision string. |
| `boolean` | `removeRedundant(RuleItem toInsert)`             Method that removes redundant rules out of the list of the best rules. |
| `static FastVector` | `singleConsequence(Instances instances, int attNum, FastVector consequences)`             generates a consequence of length 1 for an association rule. |

**Methods inherited from class java.lang.Object**

`equals, getClass, hashCode, notify, notifyAll, toString, wait, wait, wait`

**Constructor Detail**

### RuleGeneration

```
public RuleGeneration(ItemSet itemSet)
```

:   Constructor

    **Parameters:**: `itemSet` - item set for that rules should be generated. The item set will form the premise of the rules.


**Method Detail**

### binomialDistribution

```
public static final double binomialDistribution(double accuracy,
                                                double ruleCount,
                                                double premiseCount)
```

:   calculates the probability using a binomial distribution.
    If the support of the premise is too large this distribution
    is approximated by a normal distribution.

    :   **Parameters:**: `accuracy` - the accuracy value: `ruleCount` - the support of the whole rule: `premiseCount` - the support of the premise **Returns:**: the probability value

---


### expectation

```
public static final double expectation(double ruleCount,
                                       int premiseCount,
                                       double[] midPoints,
                                       java.util.Hashtable priors)
```

:   calculates the expected predctive accuracy of a rule

    :   **Parameters:**: `ruleCount` - the support of the rule: `premiseCount` - the premise support of the rule: `midPoints` - array with all mid points: `priors` - hashtable containing the prior probabilities **Returns:**: the expected predictive accuracy

---


### generateRules

```
public java.util.TreeSet generateRules(int numRules,
                                       double[] midPoints,
                                       java.util.Hashtable priors,
                                       double expectation,
                                       Instances instances,
                                       java.util.TreeSet best,
                                       int genTime)
```

:   Generates all rules for an item set. The item set is the premise.

    :   **Parameters:**: `numRules` - the number of association rules the use wants to mine. This number equals the size *n* of the list of the best rules.: `midPoints` - the mid points of the intervals: `priors` - Hashtable that contains the prior probabilities: `expectation` - the minimum value of the expected predictive accuracy that is needed to get into the list of the best rules: `instances` - the instances for which association rules are generated: `best` - the list of the *n* best rules. The list is implemented as a TreeSet: `genTime` - the maximum time of generation **Returns:**: all the rules with minimum confidence for the given item set

---


### aSubsumesB

```
public static boolean aSubsumesB(RuleItem a,
                                 RuleItem b)
```

:   Methods that decides whether or not rule a subsumes rule b.
    The defintion of subsumption is:
    Rule a subsumes rule b, if a subsumes b
    AND
    a has got least the same expected predictive accuracy as b.

    :   **Parameters:**: `a` - an association rule stored as a RuleItem: `b` - an association rule stored as a RuleItem **Returns:**: true if rule a subsumes rule b or false otherwise.

---


### singleConsequence

```
public static FastVector singleConsequence(Instances instances,
                                           int attNum,
                                           FastVector consequences)
```

:   generates a consequence of length 1 for an association rule.

    :   **Parameters:**: `instances` - the instances under consideration: `attNum` - an item that does not occur in the premise: `consequences` - FastVector that possibly already contains other consequences of length 1 **Returns:**: FastVector with consequences of length 1

---


### removeRedundant

```
public boolean removeRedundant(RuleItem toInsert)
```

:   Method that removes redundant rules out of the list of the best rules.
    A rule is in that list if:
    the expected predictive accuracy of this rule is among the best and it is
    not subsumed by a rule with at least the same expected predictive accuracy

    :   **Parameters:**: `toInsert` - the rule that should be inserted into the list **Returns:**: true if the method has changed the list, false otherwise

---


### count

```
public int count()
```

:   Gets the actual maximum value of the generation time

    :   **Returns:**: the actual maximum value of the generation time

---


### change

```
public boolean change()
```

:   Gets if the list fo the best rules has been changed

    :   **Returns:**: whether or not the list fo the best rules has been changed

---


### getRevision

```
public java.lang.String getRevision()
```

:   Returns the revision string.

    :   **Specified by:**: `getRevision` in interface `RevisionHandler`
    :   **Returns:**: the revision


---


|  |  |  |
| --- | --- | --- |
| **Overview** \| **Package** \| **Class** \| **Tree** \| **Deprecated** \| **Index** \| **Help** \| **Weka's home** | |  |
| **PREV CLASS**   **NEXT CLASS** | **FRAMES**    **NO FRAMES**     **All Classes** |  |
| SUMMARY: NESTED \| FIELD \| CONSTR \| METHOD | DETAIL: FIELD \| CONSTR \| METHOD |  |


---
